# Women’s Educating and Coping Strategies for Cultivating Supportive Web-Based Spaces for Discussing Sexual and Reproductive Health: Co-Design Study

**DOI:** 10.2196/62716

**Published:** 2025-02-26

**Authors:** Hyeyoung Ryu, Wanda Pratt

**Affiliations:** 1 Information School University of Washington Seattle, WA United States

**Keywords:** sexual and reproductive health, women’s health, stigma, microaggression, coping, counternarrative, social media, web-based communities, support, co-design

## Abstract

**Background:**

Stigma surrounding women’s sexual and reproductive health (SRH) often prevents them from seeking essential care. In South Korea, unmarried women face strong cultural taboos, increasing their risk for conditions such as pelvic inflammatory disease, infertility, and cervical cancer. While many unmarried women turn to web-based communities for support, these spaces frequently expose them to microaggressions, further discouraging their access to health care and worsening their health risks.

**Objective:**

We aimed to encourage a safe space for seeking support on the culturally taboo topic of SRH by counteracting and reducing web-based microaggressions. We sought to make these last-resort safe spaces supportive by reducing and preventing microaggressions, fostering coping strategies, and educating rather than solely punishing perpetrators.

**Methods:**

We conducted co-design sessions with 14 unmarried Korean women. In the first co-design session, we introduced the term microaggression and collaborated with participants to create base design components aimed at countering and preventing microaggressions. In the second co-design session, participants initially viewed examples of microaggression comments, then designed using the provided base design templates inspired by their suggestions from the first session and finally designed for a scenario where they would be seeking support. We analyzed co-design session transcripts using inductive and deductive methods.

**Results:**

Our analysis revealed 6 goals addressing coping strategies, educational approaches, and cultural characteristics shaping participants’ designs. Reflective coping strategies were supported through designs that numerically indicate positive support and provide holistic views of diverse perspectives, helping participants reassess provocative situations with cognitive clarity. Suppressive coping strategies were fostered by encouraging less-emotional responses, empowering participants to address microaggressions logically without self-blame. Educational approaches emphasized fostering shared awareness of microaggressions and providing respectful education for perpetrators about the harm their words can cause. Participants suggested counterspeech mechanisms, including rephrasing suggestions and public educational resources, to balance education with freedom of expression. They also proposed that forum-approved experts guide discussions to ensure accurate, empathetic responses and support users in addressing nuanced situations effectively. Cultural characteristics heavily influenced these goals. Participants noted the nebulous nature of microaggressions, their reluctance to burden their social support network, and societal perceptions of women as overly emotional—all of which shaped their desire for designs that enhance logical justification. For example, participants preferred tools such as expert-led discussions and comprehensive perspectives to rationalize their experiences while reducing stigma.

**Conclusions:**

Our work advocates for prioritizing educational and explanatory approaches over punitive detection and deletion measures to create supportive web-based spaces for individuals discussing stigmatized SRH. By integrating culturally informed coping strategies, counter speech mechanisms, and educational designs, these tools empower microaggression targets and allies while fostering reflection and behavior change among perpetrators. Our work provides a first step toward counteracting microaggressions and ultimately encouraging women to seek the needed SRH care.

## Introduction

### Background

In many cultures, including South Korea (hereafter referred to as Korea), unmarried women grapple with the stigma surrounding sexual and reproductive health (SRH), often opting to suffer in silence rather than seek necessary care [1-6]. Conversations about SRH topics—such as contraception, menstruation, sexual discomfort, or sexual pleasure—remain taboo, hindering access to essential services, such as screening and prevention of sexually transmitted infections (STIs) [3,7]. This reluctance to seek care poses substantial health risks, including pelvic inflammatory disease, ectopic pregnancy, and infertility [8]. Despite efforts to challenge traditional beliefs and decouple female sexuality from marriage [8,9], many women remain reticent to openly discuss SRH or seek related care, such as cervical cancer screening and prevention [6]. The consequences of this stigma can be dire. For example, Korean women who face many microaggressions regarding SRH [10] also experience disproportionately high rates of cervical cancer, with both native and immigrant populations being affected [6]. Despite the availability of preventive measures such as the human papillomavirus vaccine, the persisting stigma surrounding SRH care hampers efforts to address these health disparities [10]. Stigmatized SRH refers to topics considered taboo for unmarried women, such as STIs, sexual pleasure or discomfort, pregnancy, and contraception. However, the stigma also extends beyond these issues, limiting access to care for non-STI or pregnancy-related concerns, such as cervical cancer, and underscoring the broader barriers unmarried women face in SRH care.

For such stigmatized health topics, many people turn to web-based communities for support [11,12], but those web-based spaces also often contain microaggressions—communications that put down, insult, or invalidate them. These microaggressions not only harm people’s physical and psychological health [13-17] but also discourage people from seeking necessary health care [10,18,19]. This problem is particularly salient for women seeking SRH care, as it is a taboo topic in many cultures [1,20,21]. The perpetrators of web-based microaggressions include users who intentionally or unintentionally harm support seekers, including within-group perpetrators [10,15,22]. In this study, we focused on the web-based microaggressions that South Korean women face, often from other women, when seeking support for the recommended SRH care.

To jointly imagine designs that could help counteract web-based microaggressions, we co-designed with unmarried women—the targets of these microaggressions—who grew up in Korea and have used web-based forums to seek SRH care. In this study, we define the term *used* as not solely the active use of posting or commenting but also the passive use of viewing posts and comments of the web-based forum user’s interest. In addition, we define “targets” as the women who experience microaggressions related to their SRH. This definition aligns with the concept of *targets* in microaggression literature by Sue et al [23], which describes individuals who are subjected to prejudice and discrimination. While the study by Sue et al [23] focused on racial microaggressions, we expanded the term to include gender-based prejudice and discrimination.

In this study, we focused on designing solutions to counteract web-based microaggressions to cultivate a resilient and safe space for support on stigmatized SRH. We sought to make these last-resort safe spaces truly supportive by reducing the negative effects of microaggressions on the targets and allies’ health and well-being and educating rather than solely punishing perpetrators. We refer to allies as individuals who challenge bias within their own communities and actively support others, even within marginalized groups [24,25]. Examples include anonymous women users in web-based health communities, as well as friends who, while also facing stigma related to SRH, work to counteract the microaggressions they experience or witness others experiencing.

Our work focuses on presenting the findings from 2 co-design sessions with unmarried Korean women. We identified 6 goals participants had for counteracting web-based microaggressions to create a resilient and safe space for seeking and providing support on stigmatized SRH. On the basis of these 6 goals, we uncovered that, unlike most web-based content-moderation approaches that focus on detecting and deleting offensive content, most women would prefer systems that help them cope with web-based microaggressions and help perpetrators reflect on the ramifications of their words. In this paper, we have contributed designs that support these goals and preferences.

### Related Work

In this section, we connect with 3 bodies of work related to our research: rethinking content moderation, supporting vulnerable users’ coping, and designing without compromising vulnerable users’ agency.

#### Rethinking Content Moderation: The Shortcomings of Detection and Deletion Approaches

The predominant content-moderation strategy on platforms such as Facebook and Twitter (subsequently rebranded X) heavily relies on detection and deletion of harmful content [26-28]. These methods focus on removing content after it has been flagged, which makes them predominantly reactive. This approach can leave users feeling unsupported and frustrated, especially when it comes to content that falls into a “gray area,” such as microaggressions, that may not explicitly violate platform policies but still causes harm [29,30].

Ma et al [30] highlight this gap, noting that the current detection and deletion model fosters an adversarial relationship between users and platforms, where moderation feels punitive rather than supportive. Myers West [31] also emphasizes the need for more nuanced moderation practices, particularly in ambiguous cases where platform guidelines are unclear, stressing the importance of building a culture of responsible web-based citizenship that goes beyond mere content removal. Similarly, Sharevski et al [29] demonstrate the ineffectiveness of warning tags and labels, which often fail to engage users meaningfully or prevent the harm caused by problematic content. These limitations underscore the weaknesses of the detection-first approach.

Despite advancements in moderation technologies, the focus remains on swiftly identifying and removing harmful content [32-35]. This method overlooks deeper issues of user discontent, as Jhaver et al [32] report, where users express frustration over content removal without clear explanations or guidance, often feeling that their content was unjustly deleted. These issues demonstrate that the current detection and deletion approach may exacerbate tension rather than address the root causes of harmful behavior [36-38].

In this study, we aim to address these gaps by exploring alternatives to detection and deletion, focusing on strategies that offer more nuanced, supportive solutions to handling harmful content—particularly in complex cases such as microaggressions.

#### Supporting Vulnerable Users’ Coping

The current narrow focus on detection and deletion often results in harm to the very communities that we are trying to support or protect [39,40]. For instance, Thach et al [39] demonstrate how the invisible process of web-based content moderation makes marginalized social media users face disproportionate content moderation and removal (eg, computational model could flag a person of color’s post about racial justice or racist experience as hate speech). They state that the content removal or banning of the poster from the web-based community would bring further marginalization of the already marginalized users. Thus, in an attempt to better support and protect marginalized social media users, researchers from the University of Michigan School of Information developed Online Identity Health Center—a website that helps marginalized users (1) understand different social media platforms’ policies and rules about content takedowns and (2) provide easy-to-understand resources on different social media guidelines and what to do if your content is taken down [41].

In support of alternative approaches that extend the web-based content-moderation task beyond detection and permanent removal, studies on dealing with the negative consequences of microaggressions [14,33-36] have also emphasized the importance of coping in reframing web-based content moderation to better support marginalized users. Coping with negative emotions and stress induced by microaggressions is crucial to reducing the negative effects on the targets’ health and well-being [14]. Microaggressions are perpetrated and experienced subtly and often unconsciously; the target often questions the reality of the oppression [42,43]. Thus, targets of microaggressions frequently blame themselves for being overly sensitive and dismiss, if not excuse, the behavior of the perpetrators. Targets of microaggressions also show a pattern of quietly shouldering the impact of such behavior, sometimes out of fear of retaliation from the perpetrator or others involved. They also worry about reinforcing negative stereotypes (eg, women are emotionally needy and overly sensitive) if they choose to confront the perpetrator [44]. Targets of microaggressions often experience negative impacts on their well-being (eg, feelings of guilt, embarrassment, shame, regret, and remorse) after unconfronted microaggressions. Furthermore, internalizing these feelings has long-lasting effects on the target’s self-confidence and mental and physical health [45,46].

In contrast, if targets or allies choose confrontation, they must take on additional risks for themselves. Targets frequently need to circumvent fears of either being further invalidated or reinforcing negative stereotypes about their group if they were to confront microaggressions. Empirical evidence supports this notion; targets report that using confrontation opened them up to further microaggressions (eg, being further invalidated and being called “oversensitive” and “paranoid”) [47,48]. Thus, coping is essential for targets and allies in dealing with microaggressions.

Coping can be carried out through 2 emotion regulation strategies: cognitive reappraisal and expressive suppression [49,50]. Cognitive reappraisal refers to re-evaluating the meaning of a given situation to reduce its emotional impact [49,50], whereas expressive suppression refers to inhibiting an emotional response [49,51]. Although suppression may help a person avoid undesirable interpersonal consequences that can follow from expressing negative emotions, it has generally been found to be ineffective in reducing the negative emotions themselves [52-55]. Thus, Juang et al [51] suggested combining the use of both emotion regulation strategies when people encounter microaggressions. With the combined use, they indicated that expressive suppression will help in avoiding further negative interpersonal interactions, and cognitive reappraisal will help ease the emotional burden of denigration. This combined use is further corroborated by the study of Hernández and Villodas [56], where the sole use of reactive and suppressive coping was discouraged. Only reflective coping (ie, which relates to cognitive reappraisal) was associated with more positive mental health, whereas reactive and suppressive coping (ie, which relates to expressive suppression) were associated with poorer mental health after experiencing microaggressions [56]. Thus, in this study, we aimed to design features that help counteract microaggressions and do not merely detect and delete content but rather support people in reflective coping.

#### Designing Without Compromising Vulnerable Users’ Agency

The population we are designing for are microaggression targets within an oppressive social structure, where support for SRH care is difficult to find [10]. Thus, understanding how to design within oppressive social structures without compromising the agency of vulnerable users was pivotal to our work. Sultana et al [24] conducted a study on designing within a patriarchal society and investigated the economic, social, and cultural challenges faced by rural Bangladeshi women. They then presented 2 user-centered strategies for design to empower the vulnerable users. The first strategy was to try to empower women within the structures of their society, instead of trying to destroy those structures [24]. This strategy was also supported by Alsheikh et al [57] who suggested designing for openness, which supports Islamic feminist values that posit women as having agency but also operating under a set of Islamic cultural assumptions. They defined supporting agency as allowing women in Arabic culture to enact particular cultural roles in the context of their relationships rather than designing for Western feminist values (eg, demanding equality) or the patriarchal values underlying the Arabic sociocultural context.

The second strategy was to shift focus from the problems that women face to the tactics they already use to cope and look for opportunities to support these tactics [24]. Rabaan et al [58] resonated with this strategy and emphasized the importance of understanding the interwoven complexity of the sociocultural dimensions of the problem, considering historical developments, and accepting and respecting the different choices people make while understanding why they make them. With an in-depth understanding of the scarce structural support in protecting Saudi women from domestic abuse, they uncovered that resiliency was a valuable strategy for these women to sustain moving forward and even to take action to end the violence. To design for a customized interactive system that supports resiliency, Rabaan et al [58] suggested designing a screening tool to measure where a woman is at in her options, resources, needs, and concerns and what she perceives as expected or abusive.

In this study, we incorporated the 2 strategies into our design considerations. First, we designed within existing structures using the web-based forums that unmarried Korean women often use to not only seek SRH care support but also reflect the oppressive social structure in Korea [10]. Second, we focused on the tactics that unmarried Korean women already use to cope and looked for opportunities to support those tactics.

## Methods

In this section, we describe our study’s ethical considerations; co-design process, which includes co-design session 1—the creation of a base design template for improvisational enactment and co-imagination of new ideas—and co-design session 2; and co-design session analysis process.

### Ethical Considerations

This study received an exempt status from the University of Washington Human Subjects Division under Category 2, as it involves only interviews with data recorded in a manner that either does not allow for participant identification or poses no risk of harm if disclosed. The institutional review board ID is STUDY0013885. This co-design study received institutional review board approval from the University of Washington. Informed consent was obtained from all participants before their involvement in this study. Participants were provided with detailed information about the study’s purpose, procedures, potential risks, and benefits, as well as their rights to confidentiality and the option to withdraw from the study at any time without penalty. In addition, participants were given the opportunity to ask questions and receive clarification before providing their consent. All study data were deidentified, with participants referred to by their ID (eg, P [participant ID number]) in co-design transcripts and session designs. Personal information, such as names and ages, was stored in a separate file. Participants were compensated with a US $25 Amazon gift card for each co-design session, totaling US $50 for their participation. To protect participants’ privacy, we ensured that no personal identifiers were linked to any images included in the manuscript.

### Co-Design Process

#### Overview

Co-design, defined by Sanders and Stappers [59] and Kleinsmann and Valkenburg [60], involves collective creativity and shared knowledge among participants, leading to a comprehensive understanding of design content and process [59-61]. This process, also known as joint inquiry and imagination, encourages ethical engagement and starts with abduction, suggesting potential solutions [61,62]. Our study used a modified approach for remote co-design sessions, aiming to stimulate creativity and facilitate discussions on sensitive topics such as SRH care [10,63]. In the following sections, we describe our participants, details for each of the co-design procedures, and our analysis approach.

#### Participant Recruitment and Consent

We recruited unmarried Korean women between the ages of 18 and 29 years, a demographic most susceptible to microaggressions around SRH care due to the assumption that they are unmarried. These younger women often seek support on the web for SRH care, and we wanted to capture a holistic understanding of the challenges they face. Among the participants, only 7 had sought clinical SRH care (eg, obstetrics and gynecology office, women’s health clinic, and public health office), while others solely relied on web-based forums. Despite these differences, both groups used web-based spaces to navigate SRH care support, so we interviewed both to inform designs that address microaggressions targeting unmarried Korean women.

Participants were required to have received 9 to 12 years of compulsory education in Korea and to be currently unmarried. Those who had received foreign curriculum–based education during their compulsory education (eg, US curriculum–based schools) were excluded from the study. Recruitment was conducted through university board flyers at Yonsei University in Korea, web-based SRH support communities for Korean women such as women-only forums in Nate Pann, and snowball sampling.

Participants for this study were recruited using a multistep process designed to ensure their informed and voluntary participation. Potential participants initially expressed interest by completing a screening questionnaire. This questionnaire was used to verify eligibility based on the study’s inclusion and exclusion criteria.

Once eligibility was confirmed, participants received a consent form via email, which provided comprehensive details about the study. The consent form outlined the study’s purpose, the inclusion and exclusion criteria, the procedures involved in participation, the potential benefits and risks, the compensation offered, and the measures taken to protect participant confidentiality and privacy. Participants were encouraged to review the consent form independently, and a minimum of 48 hours was provided for this review to ensure they had adequate time to make an informed decision.

Following this review period, we scheduled individual calls with each participant to discuss the consent form further. During these calls, participants had the opportunity to ask questions, seek clarification about the study, and address any concerns they might have. After ensuring that participants fully understood the consent form and its implications, we proceeded with obtaining their signatures. Once consent was obtained, we scheduled the first co-design session with each participant.

#### Co-Design Session 1 Procedure

We conducted co-design session 1 as contextual research to provide the necessary background for co-design session 2 (ie, support the creation of the base design components for the co-design sessions). Co-design session 1 was conducted in a web-based setting, and each session lasted approximately 50 to 60 minutes. In co-design session 1, we asked questions about who should be responsible for counteracting microaggressions and how technology could support them. As the topic of gynecological care is taboo, we told participants that they could answer for themselves or someone they know. We first asked participants about their SRH care support–seeking experiences. Then, we explained to them what microaggressions are and showed them examples of microaggressions derived from the study by Ryu and Pratt [10]. Afterward, we asked them to think of microaggression counteraction or prevention design ideas based on their web-based SRH care support–seeking experiences.

After we received participant-generated design suggestions without specific design scenarios, we asked them about specific features (eg, flagging comments, blocking content, and content alerts) derived from currently implemented measures of major social media platforms such as Facebook [26,28] and Twitter [27,28]. Furthermore, we asked them about specific situations that social media platforms have not been able to resolve, such as “gray area” cases of moderation [30]—instances where the removed content was not wildly in violation of explicit guidelines and required some interpretation [31] (eg, when the perpetrator or target is unaware of the harm caused by microaggressions). During co-design session 1, we told participants to ignore current technological or financial limitations when ideating their designs.

#### Co-Design Session 2 Procedure

**Figure 1:**
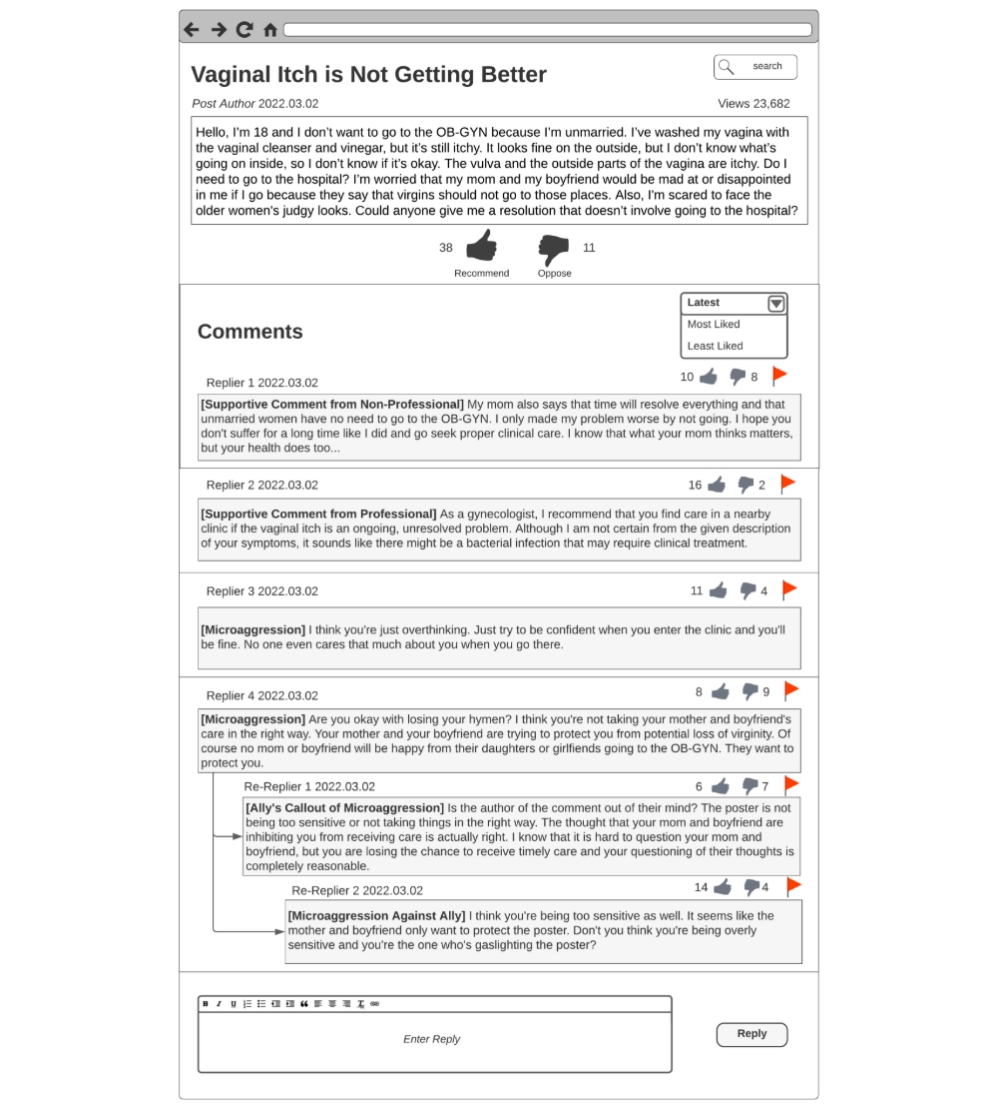
Design session sample scenario. It mimics the format and features of forums that unmarried Korean women currently go to seek sexual and reproductive health care support. The features that the design session sample scenario reflects from the original forum are the (1) post structure (ie, title, author ID, date, post views, post content, and number of post recommendations and oppositions); (2) comment structure (ie, comment filtering options and replier name, reply date, number likes and dislikes, and a comment flagging button for each comment or replies to head comment); and (3) reply and comment posting structure (ie, reply writing box and reply posting button).

On the basis of co-design session 1, we created a base design template that we showed to the participants during co-design session 2. We made a post comment sample using a hypothetical scenario with a post topic commonly seen in the web-based forums that unmarried Korean women seek SRH care support on. The hypothetical scenario focused on vaginitis and clinical visit concerns (Figure 1) because it is one of the most common concerns shared by women in web-based forums [10]. On the basis of our reviews of existing forum posts, we included comments that reflect those that showed genuine support or microaggressions from other users. We also included the ally’s response to the microaggressions and showed the microaggression perpetrators attacking the ally to reflect the current state of web-based support and microaggressions [10,42,44,64]. For the format and features (eg, post and comment like and dislike functions with the number of likes and dislikes displayed and comment filter options), we made the post comment sample reflect those of web-based forums that unmarried Korean women typically seek support from. To provide an initial design that participants could react to and provide feedback on, we presented templates for each participant-suggested design found in co-design session 1 next to the post comment sample.

Co-design session 2 was divided into three sections: (1) viewing only the post comment sample (Figure 1) without the base design template; (2) designing after viewing the base design template, which were reenactments of the participant-suggested designs; and (3) designing for a situation where the participant themself would be the support-seeking post author. Each design session was held with each participant individually using Lucidchart software (Lucid Software Inc) as the co-design tool and lasted approximately 50 to 60 minutes. Lucidchart is a design tool that facilitates remote, synchronous, and collaborative design. As our participants took part in the co-design sessions remotely, we used Lucidchart to build, replicate, and share post comment samples and design templates developed during co-design session 1. In co-design session 2, participants used Lucidchart to design web-based microaggression counteraction strategies collaboratively and synchronously.

We used the same group of participants from the initial design needs interviews because we wanted to empower them by actualizing their own designs and reflecting on not only their own but also others’ designs to counteract microaggressions. Participants received US $25 for participating in co-design session 2. We audio-recorded the interviews and used a professional transcription service to have the recordings transcribed verbatim.

We wanted to learn about the participants’ design ideas when they saw the post comment sample, so we first asked the participants to review only the post comment sample and tell us about their thoughts on how they would design the forum to counteract web-based microaggressions. Then, we asked them the same question after showing the base design template for counteracting web-based microaggressions and prototyped each design and their suggested use. We encouraged them to talk aloud as they created their designs and prompted them to explain why they wanted the microaggression counteraction feature to be designed a certain way. We asked the participants to make edits on their own but assisted them when they were experiencing difficulties.

Before and during co-design session 2, we explained that they did not have to use the design in its suggested form or at all if they did not want to use the feature. Also, we emphasized that they could alter the design to better reflect their intended use of the design (eg, adding and removing options and changing the phrases). At the end of the session, we reviewed the designed web-based microaggression tool together to check if there had been any misinterpretations and to give them the opportunity to edit their designs.

Finally, to understand how the web-based microaggression counteraction design would change if they had designed it only for themselves, we asked the participants if they would change anything if they were the ones writing the posts and receiving the microaggressions.

### Co-Design Session Analysis

A total of 2 authors participated in the analysis. The first author acknowledges her standpoint as an educated, unmarried Korean woman. She is a native Korean who received primary education in Korea and received additional intensive English language education in middle and high school. Although she is not an avid participant in the comment sections of the web-based spaces where unmarried Korean women seek support, she has investigated numerous interactions of other users, advocated for safe web-based spaces free of microaggressions, and is intrigued by the use of language to mark and protect microaggression targets. She acknowledges that her positionality influenced this study to some extent but also helped her interpret the participants’ design ideas and influences from the Korean sociocultural context. The second author is from the United States and has done extensive research on web-based health communities as well as design research with people from marginalized communities. Her cultural background has made her much more aware of microaggressions and their negative effects on her life over decades, which has helped to complement the first author’s more recent exposure to the concept of microaggressions. This complementary background also helps them consider the broader implications of this work. Because much of the second author’s research portrays the benefits of web-based health communities, she may perceive more positive possibilities for web-based health communities in this study.

The first author completed the analysis of both co-design sessions 1 and 2, with support from the second author using the ATLAS.ti analysis software (Lumivero). We analyzed 28 transcripts in total, consisting of 14 (100%) transcripts from co-design session 1 and 14 (100%) transcripts from co-design session 2. Both co-design sessions were analyzed using the same procedure. The first author cycled between field notes kept during the co-design sessions and the co-design session transcripts in an approach meant to build on themes in real time. To begin, the first author coded 5 co-design session transcripts using an open coding approach [65], labeling what she saw in the data. Then, she discussed the codes with the second author, refined these into a revised codebook, and finished coding all the remaining transcripts. Following another round of discussion and clarification, the authors finalized the codebook. As open coding was completed, the first author conducted a round of deductive coding that focused on sentences about each microaggression counteraction feature. The 2 authors then discussed interpretations and addressed any discrepancies in the application of the code set of features to counteract microaggressions and adjusted their coded data as needed. We reached saturation after coding 6 (42%) co-design session 1 transcripts and 8 (57%) co-design session 2 transcripts.

## Results

### Overview

First, we report on the participant information and participant-suggested designs and how they envisioned their designs to be enacted from co-design session 1. We further show how the designs were enacted as a design template for them to use during co-design session 2. Second, we discuss 6 goals that participants had for creating a safe and resilient web-based community that would allow them to seek support for the culturally taboo topic of SRH.

### Participants

As the co-design participants were asked to share their experiences that are not frequently or overtly discussed and are considered a sensitive topic in the culture, we used snowball sampling to recruit a total of 14 participants (Table 1); the mean age was 24 (SD 2.73) years.

**Table 1:** Participant demographics.

ID	Age (y)	Grew up in Korea	Currently living in Korea	Knowledge of microaggression before this study	Experience seeking SRH^a^ care in a clinical setting^b^
P1	24	Yes	Yes	Yes	No
P2	24	Yes	Yes	Yes	No
P3	24	Yes	Yes	No	Yes
P4	24	Yes	Yes	No	Yes
P5	23	Yes	Yes	No	No
P6	23	Yes	Yes	Yes	No
P7	26	Yes	Yes	No	Yes
P8	24	Yes	Yes	Yes	No
P9	20	Yes	No	Yes	No
P10	25	Yes	Yes	No	Yes
P11	31	Yes	No	No	No
P12	25	Yes	No	No	Yes
P13	20	Yes	No	No	Yes
P14	27	Yes	Yes	No	Yes

^a^SRH: sexual and reproductive health.

^b^For example, obstetrics and gynecology clinic, women’s health clinic, and public health office.

All participants had physical and financial access to SRH clinical services, but the women who reported “no” to seeking SRH in various clinical settings stated that they did not seek SRH care due to stigma. We explored whether there were differences in results between participants who had sought SRH clinical care and those who had not, but we found no noteworthy differences in the designs they created.

Participants who had no knowledge of microaggressions before this study elucidated that they had not heard of microaggressions, so even if they had experienced microaggressions, they were unable to name what they were experiencing. We explored whether having prior knowledge of microaggressions influenced the designs participants put forth, but we found no notable differences between those with and without prior knowledge. Participants who had prior knowledge of microaggressions had only a basic understanding, primarily from skimming social media posts. All participants noted that they would not be able to categorize microaggression types or provide a clear definition.

### Initial Design Results

In co-design session 1, we identified the features participants wanted and whom they intended the features to be used by (Table 2).

**Table 2:** Participant-suggested features from co-design session 1.

Feature	Actor using feature
Labeling type of support	Woman seeking support^a^
Choosing percentage of microaggression to view	Woman seeking support
Alerting about microaggression content with extra step of consent to view content	Woman seeking support
Microaggression comment rephrasing suggestions	Potential microaggression perpetrator or ally^b^
Microaggression flagging explanations	Woman seeking support
Messages to microaggression perpetrators or targets	Woman seeking support and potential microaggression perpetrator or ally
Professional certification badge	Woman seeking support

^a^Post authors.

^b^Commenters.

First, to ensure that post authors receive the type of support they want, participants suggested 3 design features or functionalities that could be used while people are writing their posts. The first feature would allow post authors to label their posts with the type of support sought. Participants identified five types of support: (1) emotional support, (2) informational support, (3) women-only support, (4) expert-only support, and (5) debate. Second, participants suggested choosing the percentage of comments with microaggressions post authors were willing to view. Participants suggested the inclusion of viewing all microaggression comments (100%) and no microaggression comments (0%) as options. Finally, inspired by the idea of content blocking on Instagram, participants wanted a microaggression content alert to initially block comments with microaggressions but allow people to view the comment if they click the button to view the comment.

To reduce the microaggressions received and to educate people writing comments, participants wanted support for people while they were writing comments. Participants envisioned features that would suggest rephrasing a microaggression with explanations for the suggestions. They either wanted the whole comment or specific parts of the comment to be highlighted. For the highlighted parts, participants wanted features that would provide rephrasing suggestions that could be applied with a click of a button. Some participants also wanted features that would provide the comment writer with explanations for why the rephrasing suggestion would make their comment not a microaggression. Other participants did not want a feature with rephrasing suggestions because they wanted to protect comment writers’ freedom of speech.

Participants suggested three types of features to identify microaggressions in comments for the original poster, commenters, and readers after a comment has been posted. First, participants wanted to be able to flag comments that contain microaggressions along with an explanation for why it was a microaggression. Participants explained that the current social media platforms allow them to report or flag comments but that their choices for indicating why a comment should be flagged are too general to help commenters understand why their comments contained microaggressions. In particular, participants wanted the flagged reason to be shown in the public explanations for each flagged comment, which would help the community and the commenter learn about microaggressions. Second, participants wanted features that would send messages to the microaggression perpetrators or the targets of the microaggressions—who could be either the original poster or another commenter. To provide reassurance and validation of the targeted person’s thoughts and feelings, participants wanted to send messages letting them know that the microaggressions were noticed. For the perpetrators of the microaggressions, participants thought that perpetrators might continue to write microaggressions if they were merely given warnings that their comments had been flagged. However, participants were uncertain about the exact content they would include in such messages or the flagging explanation, and the interviews did not include time for them to think through and compose a sample. Thus, in the design session, we provided an opportunity for participants to compose their explanations or messages if they wished to.

As a justification for their final post comment design suggestion, participants wanted health professionals to have more accountability for their words because the participants placed higher trust and reliance on these professionals. Participants suggested a feature that would flag comments from health professionals. Participants wanted health professionals to make their status as a health professional public, thus putting their status and reputation on the line and making them more responsible for what they say. Participants wanted to create a professional certification badge based on the badges stamped next to the professionals’ replies in Naver Jisikin—a widely used question-and-answer forum in Korea.

**Figure 2:**
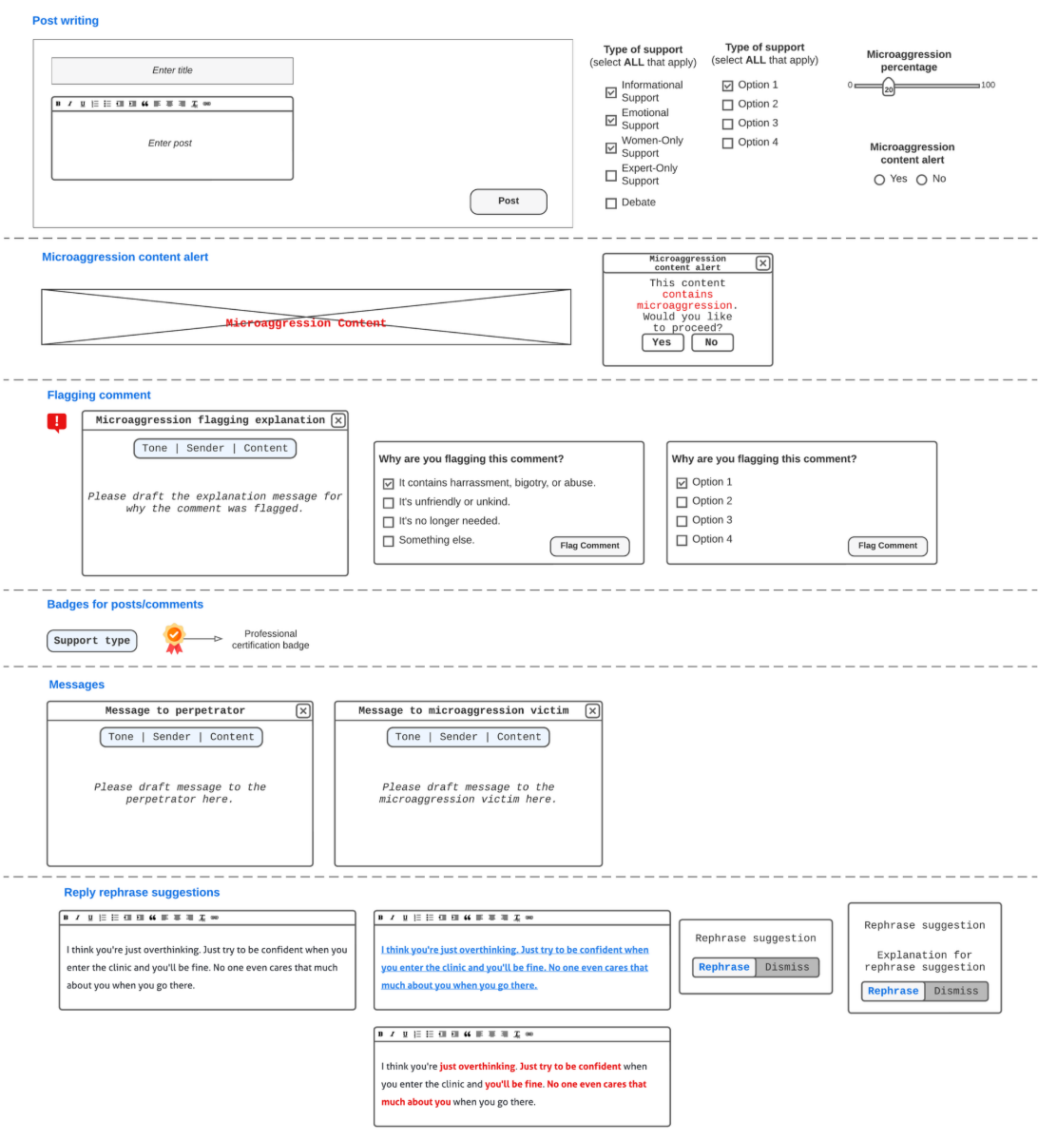
Co-design session 2 base design template. It shows the set of participant-suggested designs found in co-design session 1. For support during post writing, participants had the option to design with labeling the type of support post authors needed (a-1) which would be shown with the support type badge on the post (a-2), microaggression percentage (b), and microaggression content alert (c-1) which would be shown on the comments as (c-2). For during comment writing support, participants were able to design reply rephrase suggestions by highlighting the rephrase needed parts (g-1) and offering the rephrase suggestions with or without explanations (g-2). For support after a comment has been posted, participants could choose to implement the professional certification badge (e) and design the flagging options (d-1), flagging explanation messages (d-2), and messages to microaggression targets and perpetrators (f).

With the participant-suggested designs and their descriptions of how they should be envisioned, we made templates for each design (Figure 2). These designs allowed participants to experience their designs’ enactment in co-design session 2.

### Design Goals

#### Overview

Through the analysis of the co-design sessions, we found that participants had 6 goals in counteracting web-based microaggressions to create a safe and resilient web-based community for seeking support on the stigmatized SRH. These goals arose in co-design session 1 but were further expanded upon in co-design session 2 after they had learned more about microaggressions and were able to tangibly interact with their envisioned designs. We first describe each of the goals and participants’ design ideas for achieving these 6 goals. The figures of the design ideas shared in this paper were created by the participants using Lucidchart software.

- Goal 1: having shared knowledge of microaggressions among all users.
- Goal 2: indicating positive support numerically.
- Goal 3: responding with less emotion to microaggressions.
- Goal 4: having forum-approved experts guide the discussions.
- Goal 5: respecting perpetrators’ freedom of speech while educating them to understand their words' negative impact.
- Goal 6: knowing the “reality.”

Furthermore, we describe how the 6 goals were affected by 3 cultural characteristics: the nebulous nature of microaggressions, reluctance to burden their social support network, and questioning the validity of their own thoughts due to the social perception that women are too emotional.

#### Goal 1: Having Shared Knowledge of Microaggressions Among All Users

**Figure 3:**
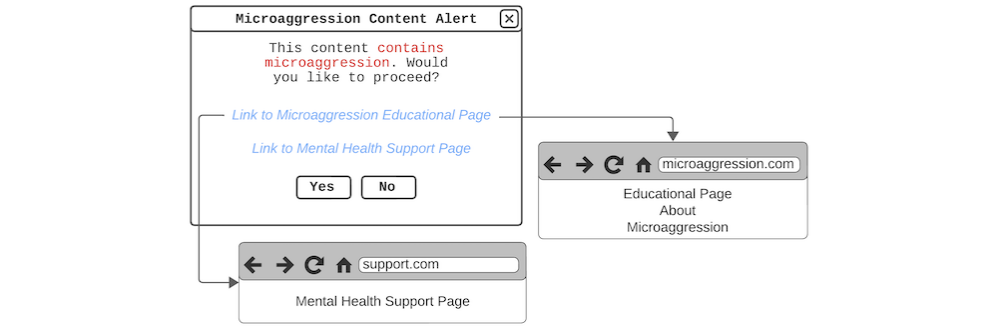
Microaggression content alert design by P6. On the microaggression content alert, links to a mental health support page and an educational page about microaggression are added.

Participants advocated for providing people with educational resources about microaggressions and supporting people harmed by microaggressions. They wanted unmarried Korean women post authors to be more informed about microaggressions by adding links to microaggression-related educational resources and support groups under the microaggression content alert option (Figure 3):

First of all, we have to understand that there’s a lack of shared knowledge here. People don’t know what microaggressions are. How are we supposed to defend ourselves without knowing what to defend ourselves from? When we see the [microaggression] content alert, we need to have additional resources or groups we can access to get support or a better understanding of microaggressions.

P3

**Figure 4:**
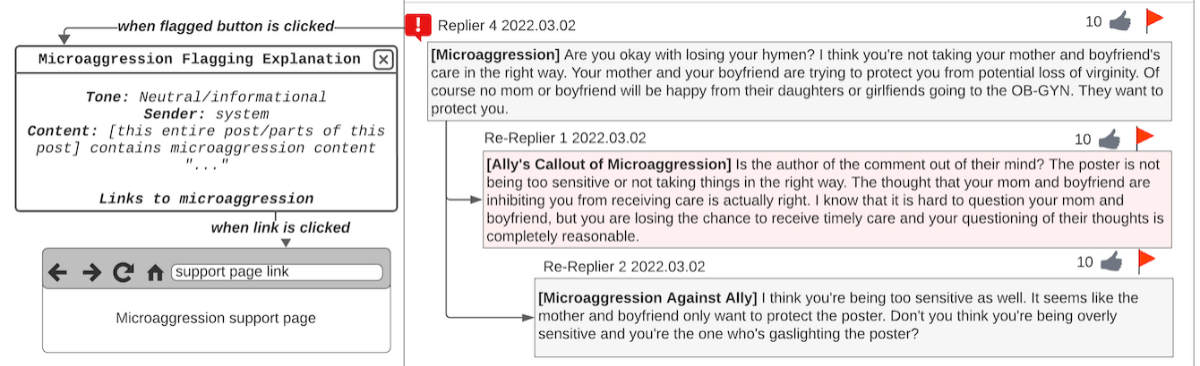
Educational flagged reason explanation design by P11. When the flagged button next to a flagged comment is clicked, the user can see a message that the comment contains microaggression content and links to microaggression support pages.

Beyond the post authors, participants also wanted other web-based forum users to learn about microaggressions and their impacts. Thus, participants suggested adding microaggression educational resources to explanations next to flagged comments (Figure 4):

It’s not just people who post on the [online] forums that need to know about microaggressions. Other people using the [online] forums also do too. I think the best way to do that is to have information [about microaggressions] or [website] links to microaggression information right next to the comments, the ones that are flagged, so everyone can see it.

P11

**Figure 5:**
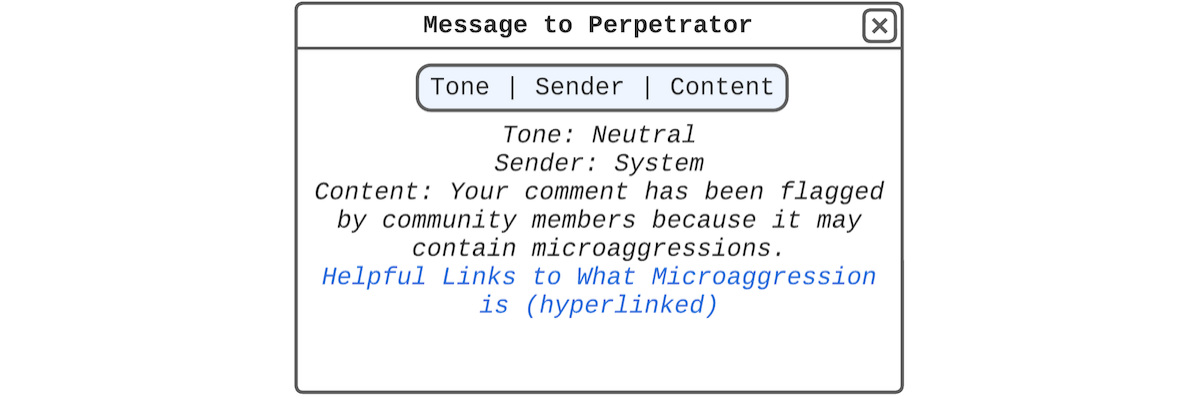
Message to perpetrator design by P8. The message to the microaggression perpetrator is designed to have a neutral tone and shows the message that the perpetrator’s comment has been flagged because it may contain microaggressions. The message also has links to help the perpetrator understand what microaggressions are.

Furthermore, they wanted to ensure that perpetrators were educated on microaggressions by sending them a private message with additional microaggression educational resources attached to the explanation of why the comment was flagged (Figure 5):

I don’t want the so-called perpetrators to be confused and be like what the f***? when they get a message after writing something that they think in their head is supportive. I don’t want them [perpetrators] to be mad. I want them to understand. Having the message [sent to microaggression perpetrators privately] would point them to the right direction of understanding their “unintentional” wrongdoing.

P4

#### Goal 2: Indicating Positive Support Numerically

**Figure 6:**
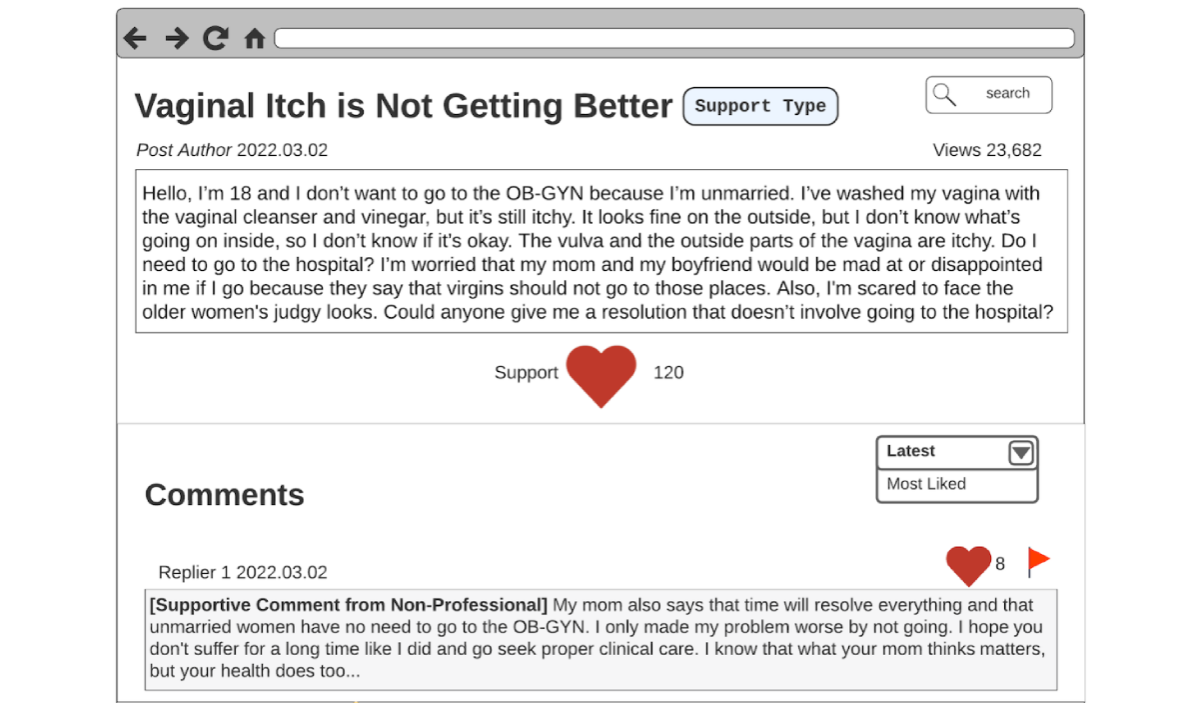
Support button design by P13. Instead of the like and dislike button, only the support button was made available with a heart icon to indicate support. The number of people who pressed the support button was displayed next to the support button.

Participants advocated for emphasizing positive support through numerical indicators and deleting negative support indicators, such as thumbs-down or dislike options for posts and comments. They suggested transforming the like button into more supportive options (eg, text forms: helpful supportive; icon form: heart; Figure 6):

Why does the like button have to be a thumbs-up button? I think it could be words like “supportive”. It shows more of why people clicked the button. People have to put in another layer of thought before pressing the button. They would have to think like “Oh was this actually supportive?”

P8

All participants except one suggested the number of clicks on the transformed like button to be displayed next to each post or comment (Figure 6). With the number of likes, participants stated that they could organically find the comments most people found most supportive, which they would also likely find helpful:

Numbers are produced naturally. That sounds weird saying it out loud but it’s something you can’t refute. The more numbers, the more support (for the comment).

P6

As participants perceived that the numerical indicators would be helpful in finding truly supportive comments, many of the participants did not choose to send the messages to post authors who have faced microaggressions in the comment sections. Participants thought that sending the messages to post authors would be “unnecessarily coddling” [P6] when they could be given the agency to learn how to sort out truly supportive comments on their own with the help of numerical indicators:

I want to give women the strength to defend and sort out helpful information on their own. I don’t know if I would describe the internet as a jungle, but it sometimes is. You got to know what’s poison and what’s not. Like the color of mushrooms, the brighter they are, the more poisonous they are. The numbers [of support] next to a comment is the color [of mushrooms].

P1

#### Goal 3: Responding With Less Emotion to Microaggressions

**Figure 7:**
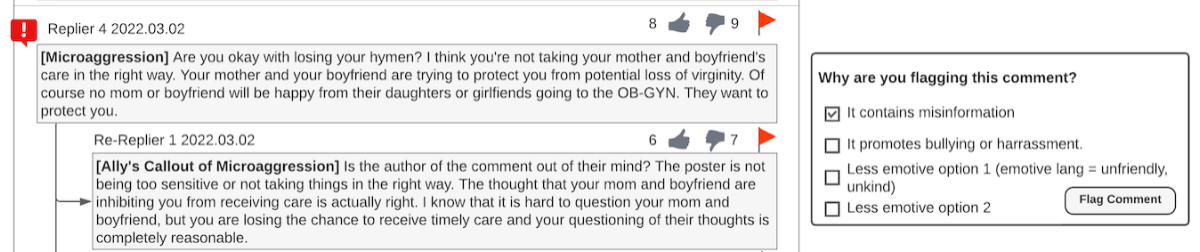
Less-emotive flagging option design by P2. Less-emotive flagging options (eg, it contains misinformation and it promotes bullying or harassment) were used to replace the more emotive current flagging options (eg, it is unfriendly and it is unkind).

After evaluating widely used flagging options in social media, participants perceived the currently used flagging options to be too emotional. Participants stated that emotive language, such as unfriendly or unkind, is too subjective and suggested changing it to less-emotive words (eg, it contains misinformation and it promotes bullying or harassment; Figure 7):

Words like unfriendly, unkind are too whatever you make of it. Microaggressions are already subjective or at least in how I understood it [microaggressions are subjective]. I think it amplifies that subjectiveness in the flagging explanation if we continue with the emotive language. We need to be less emotive using phrases like “It promotes bullying or harassment.”

P5

Participants who wanted private messages to be sent to microaggression perpetrators thought that using a neutral tone stating why the comment was flagged would be sufficient. Participants did not think a more threatening or emotionally intense response would be needed. They stated that guiding microaggression perpetrators to the microaggression-related educational resources and providing opportunities for microaggression perpetrators to learn the impacts of their microaggression comments on their own would be more valuable than penalizing them:

Neutral [tone for the private messages sent to microaggression perpetrators] is good. We don’t want to threaten them. What if they retaliate? I don’t want them to keep causing harm. We want them on our side. In that case, opening their eyes to what they are not seeing is better. Make them learn more and realize on their own.

P14

#### Goal 4: Having Forum-Approved Experts to Guide the Discussions

**Figure 8:**
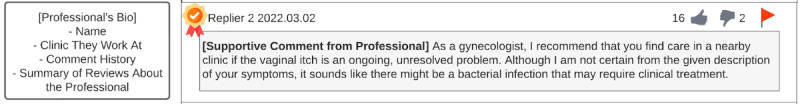
Professional certification badge and information page about professional design by P4. Professionals who are certified by the forum would have a badge next to their names when they post a comment. When other users click on the professional’s ID, they would be able to see a hovering information page with information about the professional’s name, the clinic they work at, comment history, and a summary of reviews about the professional.

Participants wanted experts to help provide guidance in sorting out helpful comments since they expected the forum-approved experts only to post helpful and truthful comments. To ensure that the forum-approved experts were posting only helpful and truthful comments, participants requested web-based forums to include the process of professional certification in the forum description page. In addition, they requested more information about the forum-approved professionals, such as their names, the clinics they work at, their comment histories, and a summary of the reviews about the professionals on their user page (Figure 8). With this information, participants wanted forum-approved experts to help guide users to disregard comments with microaggression as well as comments containing misinformation:

People can lie about being professionals and go around spreading misinformation, so for people who are seeking support, the badge would be helpful to differentiate between certified and non-certified professionals.

P2

**Figure 9:**
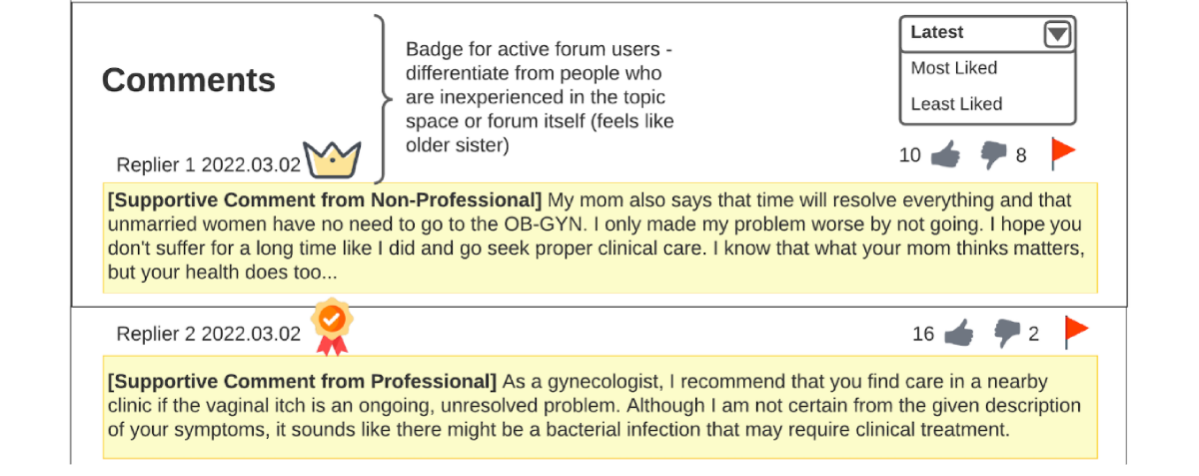
Active or experienced user badge design by P9. Active or more experienced users who could act as the older sister guide for less experienced users were identified by the forum with a crown badge.

Participants also requested certifications and badges for active forum users (Figure 9). Participants wanted the active forum users to be differentiated from people who were inexperienced about the topic or new to the forum. They wanted the active forum users to act as the “older sister” [P9] role by guiding the discussion in the right way for topics they were more knowledgeable about. With both types of certified experts, participants thought that microaggression comments that are disrespectful and contain misinformation could be weeded out and counteracted:

Kind, emphasis on the kind, experienced, or more knowledgeable users should be differentiated from people who are inexperienced in the topic space or forum itself. They know how to deal with people who are acting disrespectfully and fend them off, you know like an older sister protecting her younger sister at a playground.

P9

#### Goal 5: Respecting Perpetrators’ Freedom of Speech While Educating Them to Understand Their Words’ Negative Impact

Most participants did not think that microaggression perpetrators would be willing to change their microaggression comments when given a choice. Thus, they chose not to implement the microaggression comment rephrase suggestions:

It’s rarely incidental when people are writing microaggressions. Deep down in their hearts, they probably do really know that they are causing harm or at least that what they’re posting could be harmful. These people know what they are doing, so rephrasing suggestions or explanations for them is only helpful if they want to know.

P2

However, participants also wanted to respect the perpetrators’ and potential allies’ freedom of speech rather than add a burden on people who were merely offering help or support. They wanted microaggression perpetrators who had the potential to become allies to be less burdened by this “big brother monitoring” [P9]:

I like the idea of a little fixing [of microaggressions] but having it still be optional. Something just doesn’t sit well with me if I am forcing the person writing the comment to change it before they even post it. I hope that they are smart enough to make the right decision and I want to believe that they could change the parts they want and make it less harmful on their own.

P7

**Figure 10:**
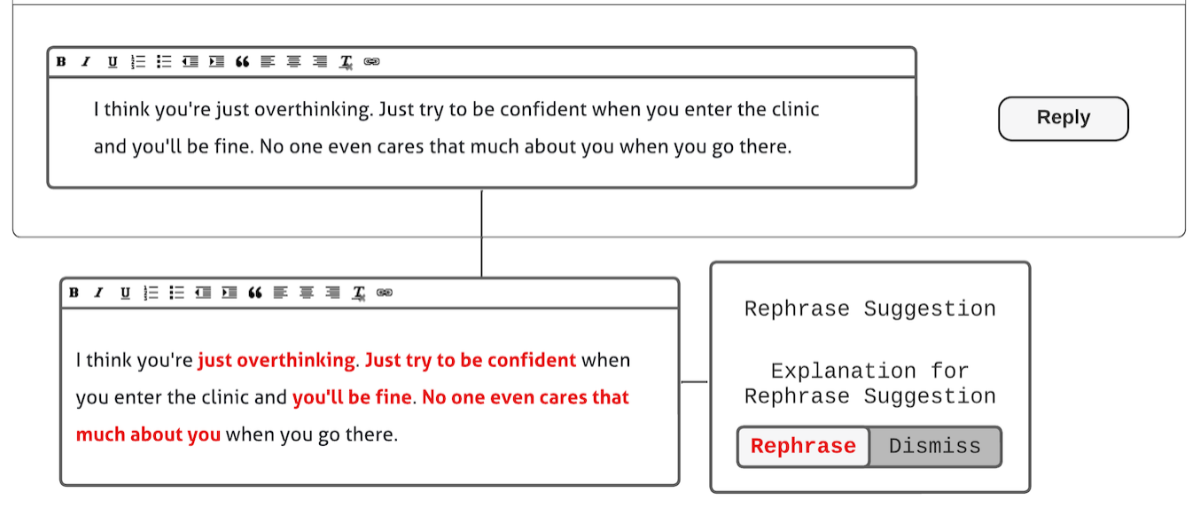
Microaggression comment rephrase suggestion and explanation design by P10. While writing the comment, the user is notified which parts of the comment contain microaggression. Then, the user receives a rephrase suggestion and an explanation for why the rephrase suggestion would make the current comment genuinely supportive. The user could choose to rephrase the comment by clicking on the “Rephrase” button or choose to not rephrase by clicking on the “Dismiss” button.

Although the microaggression comment rephrasing feature was considered an unnecessary burden by most participants, the few who wanted to provide potential microaggression perpetrators or allies the option to rephrase their comments saw potential in it. The few participants who wanted to implement the microaggression comment rephrasing feature suggested providing both the rephrasing suggestions and explanations of why the suggestions would make the comment genuinely supportive (Figure 10). These participants firmly believed that potential allies “have a good heart” [P12] and would see the value of choosing to rephrase with the rephrasing suggestion when they see the explanation attached to the suggestion:

Commenters may not have had any intention to cause microaggressions, so to further decrease microaggressions that were not intentional, having explanations [for why their comments are microaggressive and how the suggested changes make them less microaggressive] would be helpful. They don’t know the why, and people need to know the why, if there was no ill-heartedness behind their actions, to make changes to them.

P4

#### Goal 6: Knowing the “Reality”

**Figure 11:**
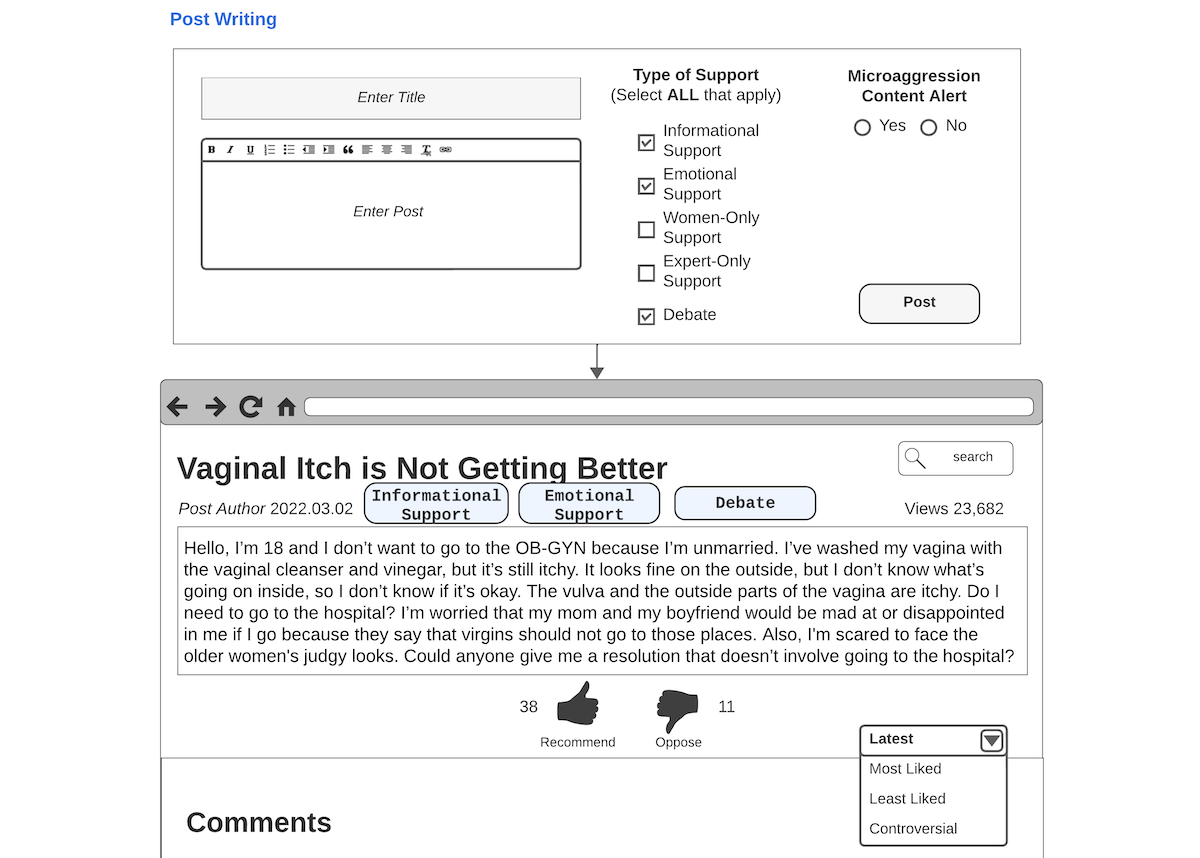
Support type label and comment sorting option design by P2. The “Debate” option was added as a type of support that post authors could indicate on their posts. In the comment sorting options, the “Controversial” option was added to see comments that had the most controversy among users.

This section is not about findings on microaggression counteraction, but rather the byproduct of participants’ evaluation of the effects of counteracting or blocking all microaggressions. During co-design session 1 ideation and at the beginning of co-design session 2, participants were focused on finding ways to counteract and combat microaggressions, as shown in the earlier subsections. However, near the end of the co-design session 2, when they were asked if there was anything they would like to change if they were the sole users of the web-based community and the microaggression counteraction features, participants stated that they had the right to know the truth or reality they were facing, even if it meant that they had to directly interact with microaggressions. Participants were concerned that showing a rosy environment where “everything is unicorn and rainbows” [P2] is deceptive and thought that users should be aware of the reality. Thus, they suggested the implementation of the debate and seeking opinions option for the support type labels and controversiality as a comment sorting option when they were designing microaggression counteraction measures for solely themselves (Figure 11):

I can honestly handle whatever it is because I want to get the whole picture – both sides, not just one cuddly side. I want to be able to face reality. It would be even better to not only have the true opinions accessible, but also easier to access. Maybe by sorting the comments here [the comment sorting button] with a controversiality sorting.

P3

The change in design for themselves showed that the participants found value in incorporating all opinions, even negative, discriminatory, and disheartening ones, to help them make a decision about their SRH care with the information gathered. However, they still wanted to have other microaggression counteraction measures put into effect, which showed that even with the features suggested to know the reality, microaggression counteraction was still deemed by the participants as pivotal and necessary:

I still want everything else [the other microaggression counteraction features P2 applied to the design for other unmarried Korean women users] there. I still want to be protected. I just also want to know the controversial opinions to make sure that I have all the information I need to make the most right? I’m not sure if that’s the right word, but you know what I mean. The most optimal decision.

P2

#### Cultural Characteristics Affecting Microaggression Counteraction Goals

**Figure 12:**
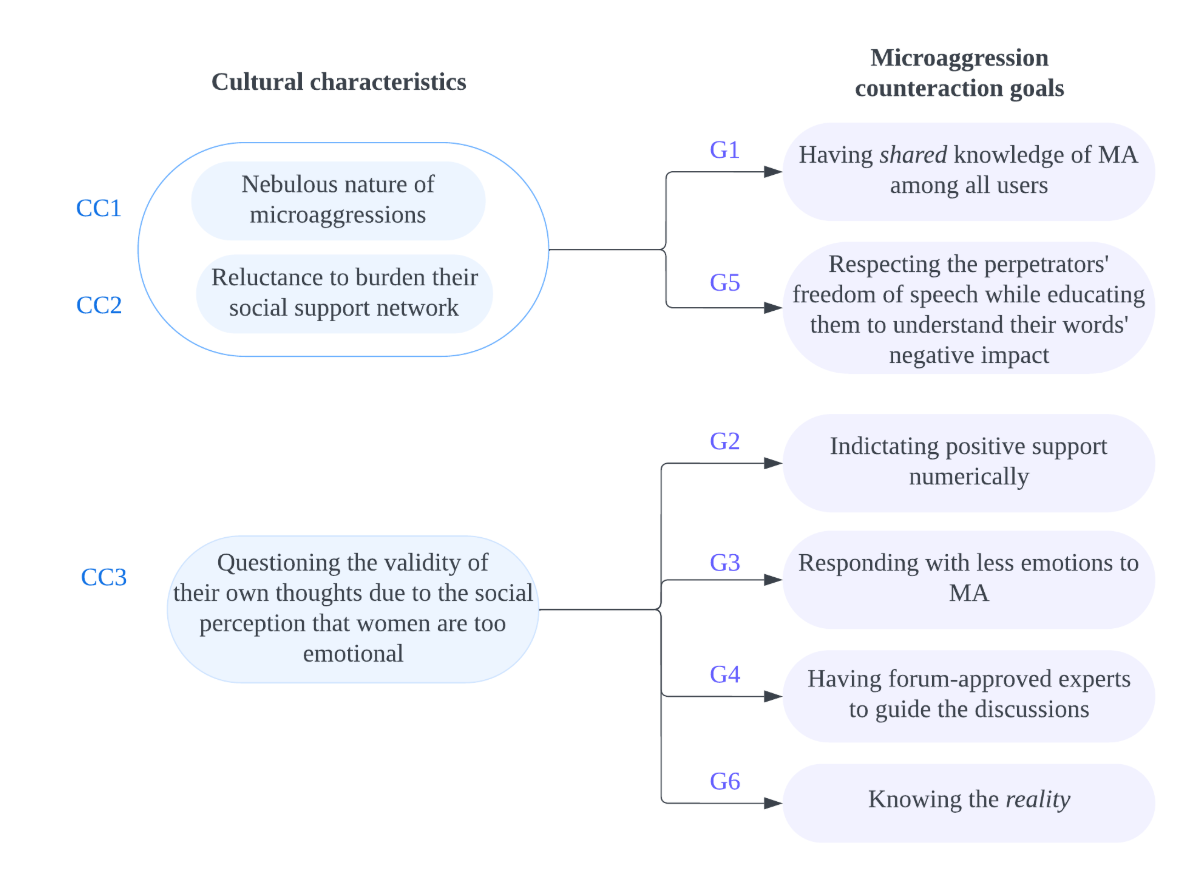
Cultural characteristics (CC) affecting microaggression (MA) counteraction goals (G). CC1 is that the microaggressions are nebulous in nature, particularly in the South Korean culture, where the language does not even have a term for microaggression. CC2 is that participants rejected approaches that would burden their social support network. CC3 is that participants questioned the validity of their own thoughts due to the social perception that women are too emotional and sensitive. CC1 and CC2 jointly influenced G1 and G5. CC3 influenced G2, G3, G4, and G6.

We uncovered cultural characteristics that affected the 6 microaggression counteraction goals and participants’ designs and ideas to support those goals. First, microaggressions are nebulous in nature, particularly in the South Korean culture, where the language does not even have a term for microaggression. Second, participants rejected approaches that would burden their social support network. Third, participants questioned the validity of their own thoughts due to the social perception that women are too emotional and sensitive. The first 2 cultural characteristics jointly influenced goals 1 (ie, having “shared” knowledge of microaggressions among all users) and 5 (ie, respecting the perpetrators’ freedom of speech while educating them to understand their words’ negative impact). The third cultural characteristic influenced goals 2 (ie, indicating positive support numerically), 3 (ie, responding with less emotion to microaggressions), 4 (ie, having forum-approved experts guide the discussions), and 6 (ie, knowing the “reality”; Figure 12).

During the discussions for goal 1 (ie, having “shared” knowledge of microaggressions among all users), participants brought up that they did not want to burden their social support network, but due to the nebulous and subtle nature of microaggressions, they were inclined to have a shared knowledge of microaggressions among all users:

You might not know if you have just said already harmful words in your daily life, so I don’t think it’s a very terrible thing. Maybe it depends, but I don’t think it should be taken that seriously. So it’s okay to give some experience and time for people to get through the flags and learn through all the comments that they said that are harmful without removing them. Support the women who could be supporters!

P7

Participants were concerned that acquiring the knowledge of what microaggressions are and how they affect the targets would be cumbersome for male perpetrators who were not interested in putting in the time and effort to see from the target’s perspective:

I would give all of them [male microaggression perpetrators] vaginas and uteruses and have them go get a pap smear done or understand the process of seeking SRH care so they can become more empathetic towards the situations and experiences that women go through every day.

P6

However, they thought that in the long term, it would help their social support network (ie, fellow female users who have the intent to help them) be less burdened to provide the support they were trying to offer:

This microaggression thing is hard to pinpoint and I just don’t want to make everyone who is trying to help feel like it’s too hard to help. I think that having a communal sense of knowledge would be better. I know that it’s going to burden women who are only trying to help at first, so I’m kind of iffy about the idea, but I think in the long term, it would help much more [to counteract microaggressions].

P7

While ideating the design ideas to support goal 5 (ie, respecting the perpetrators’ freedom of speech while educating them to understand their words’ negative impact), participants further reflected on how making judgments about users with supportive intentions based on microaggressions which are so subtle in nature would encumber users who were merely trying to be supportive. They elucidated that in the Korean culture, support is sometimes provided in the form of hard-hearted advice rather than a warm and sympathetic embracement, which would often be associated with microaggressions. Although most participants did not know what microaggressions were before this study, they acknowledged that the current delivery of support is not ideal and could be ameliorated:

You know how we [Koreans] can be. Words of affirmation are definitely not our [culture’s] love language. We [Koreans] give it to you straight and don’t want to just tell you things that will make you feel better at the moment. Although it might sound uncaring, giving you that push to snap out of it and solve your problems is our way of showing that we care. Though I agree that it doesn’t always make things better. It sometimes just hurts, and it could be [delivered] better.

P6

During the discussions for goals 2, 3, 4, and 6, participants constantly stated that sociocultural context shaped them to question their own thoughts and depend on others’ judgments to determine what is right for them. However, the same design-influencing cultural characteristic affected the designs of each goal-supporting features in different ways. For goal 3 (ie, responding with less emotion to microaggressions) supporters, the need to respond with less emotion to microaggressions stemmed from them wanting to be treated less as an emotional or sensitive person and their want to be treated as a person who can justify their opinions or decisions with logic:

[Unmarried Korean] women are just framed to be these emotional beings who are just too da** sensitive. In our society, whenever women bring up discomforts or problems, we’re told that we’re being sensitive and somewhat irrational, even by other women. I think we need the preset options for why a comment is a microaggression to be as coherent and rational as possible to help our case.

P13

Similarly, goal 2 (ie, indicating positive support numerically) and goal 6 (ie, knowing the “reality”) advocates indicated unmarried Korean women users need to be more logical in their justifications of their thoughts. However, goal 2 and goal 6 supporters had different opinions on what was required for logical justifications. Goal 6 proponents claimed that to provide a logical rationale, they needed to comprehend the diverse perspectives that other users hold to better defend themselves:

I need to know what I’m dealing with as a whole to better understand how I should be taking something. Without the whole picture, how do I know that I am making a sensible judgment of what I am reading and the people who are writing what I am reading? After I see the complete picture, I will know who I agree with more and who I agree with less.

P9

On the other hand, goal 2 supporters argued that logical rationales for one’s own thoughts could be supported by the number of support that a comment or claim has. Participants who advocated for goal 2 claimed that showing the number of negative responses to a comment would only make the questioning of their own thoughts worse rather than help them get the evidence they need to make themselves not be seen as overly emotional people:

I am uncomfortable with the idea of just having two options (thumbs-up button and thumbs-down button). Well, I’m more uncomfortable with the thumbs-down button. Seeing the number of thumbs-downs would just make me feel uneasy when I’m trying to make sure that what I’m thinking is right. It would just make me question my decisions when I don’t have to. I shouldn’t have to [question my decisions], but it’s making me do that.

P8

In contrast to goals 2, 3, and 6 advocates, goal 4 (ie, having forum-approved experts guide the discussions) proponents expanded the process of finding logical justification for their own thoughts to include the opinions of verified experts. Goal 4 proponents’ proposed ideas should not be evaluated as ideas that comply with the social perception that women are too emotional. Instead, they should be regarded as expanding the sources of knowledge they could use in strengthening their logical justifications, as shown in the following quote:

We just cannot do it all - not be sensitive or emotional and find all the medical, factual, and experiential knowledge. We need help, and that’s where experts, of course those who are certified, could help.

P12

## Discussion

### Overview

In this section, we elucidate how the goals identified from the participants’ co-design could be leveraged to build a more resilient and safe web-based space to discuss and seek support on SRH care issues. In the first part of our discussion, we describe how the goals that we identified from participants’ co-design activities would also support known psychological strategies for coping with microaggressions. In the second part of the discussion, we detail how participants’ goals support educational and explanatory approaches to counteracting microaggressions.

### Coping Strategies for Web-Based Microaggressions

The combined use of reflective coping (ie, re-evaluating the meaning of a given situation to reduce its emotional impact [50]) and suppressive coping (ie, inhibiting of an emotional response [51]) have been proven to be effective in coping with microaggressions [50,51,56]. Participants’ goals uncovered in the co-design sessions show how to achieve reflective and suppressive coping strategies in web-based microaggression counteraction measures.

Goals 2 (ie, indicating positive support numerically) and 6 (ie, knowing the “reality”) supported the microaggression targets to achieve reflective coping. Individuals using reflective coping styles examine causal relationships, reflect, and engage in behaviors that are intended to produce changes in their affective states and cognitive processes [66-68]. Indicating positive support numerically and seeing the whole picture by understanding both sides was suggested by participants to help them reassess the given situation with an objective standard, which is pivotal to cognitive reappraisal or picturing the provocative situation from a third-person perspective [48,50]. By understanding how many people explicitly support an opinion or argument, participants thought that they would be able to evaluate whose opinions they should trust. In addition, by seeing the whole picture and not just the positive support numerical indicators, they thought that they would not be making a judgment based on viewing only one side and be able to change their affective states and cognitive processes with a holistic understanding of the provocative situation [67,68].

For suppressive coping, participants suggested goal 3 (ie, responding with less emotion to microaggressions) with an added level of agency. In a notable study that investigated strategies to counteract web-based ableist microaggressions, the coping mechanisms for ableist microaggressions were found to align with traditional suppressive coping mechanisms (eg, deleting comments and letting it go, blocking rather than reporting, and taking a break from social media) [69]. However, in this study, we found that responding with less emotion to microaggressions did not necessarily mean to “avoid” dealing with microaggressions. Rather, suppressive coping was transformed into active responses to microaggressions but with less-emotive words. By using less emotion in response to microaggressions, either in private messages or through flagging options, participants thought their claims that a comment contained microaggression would be less subjective. Making their claim seem less subjective was pivotal because microaggression targets tend to blame themselves for being overly sensitive and worry about reinforcing negative stereotypes [10,42,44,47,48].

Thus, with tools to support the stated goals, we hope to expand the ways to incorporate reflective and suppressive coping mechanisms for the targets of web-based microaggressions. However, we advise designers to implement the participant-suggested designs with caution, especially for reflective coping–supporting designs. The goals that were identified to support reflective coping—goals 2 (ie, indicating positive support numerically) and 6 (ie, knowing the “reality”)—were influenced by the cultural characteristic that unmarried Korean women are often exposed to situations where they question their own thoughts and value the public’s account of their opinions because they are socially perceived to be overly emotional and sensitive. Thus, the implementation of tools to support reflective coping would need to be provided with constant exposure to microaffirmations: subtle verbal remarks and storytelling microaggression targets engage to affirm each other’s dignity, integrity, and shared humanity [70]. Jones and Rolon-Dow [38] support the notion that constant exposure to microaffirmations helps targets understand what microaggressions and microaffirmations are, how they are perpetuated, and how they impact the target. With the benefits of repeated listening to microaffirmations, women would be able to achieve deeper reflective coping [38,70,71]. Targets of these microaggressions would be able to better construct a narrative about the microaggressions they experienced and develop more effective action plans to change their affective states and cognitive processes [38,71].

### Educational and Explanatory Approaches to Counteracting Microaggressions to Increase Awareness of Microaggressions Among All Users

Goals 1 (ie, having shared knowledge among all users) and 5 (ie, respecting perpetrators’ freedom of speech while educating them about the negative impact of their words) can be achieved by using approaches that focus on educating people about microaggressions and their harmful effects, as well as explaining why certain comments are considered microaggressions. By highlighting the harmful consequences of microaggressions, such approaches can help perpetrators evaluate the ramifications of their comments and reconsider causing future harm.

Explanations of what microaggressions are and their harmful impacts make current perpetrators aware of the problem and provide them with a chance to become proper allies by reexamining their own and others’ roles and responsibilities [25,31,32]. Potential allies or perpetrators, especially those of the same gender who are experiencing similar oppressions or have overcome them, need to be more aware of their roles and responsibilities in reducing web-based in-group microaggressions. When people normalize oppression rather than acknowledging it, they unintentionally reinforce it with further microaggressions [10,22]. In contrast, when those who have overcome oppression offer empathy, support, or acknowledgment of the harm, they help others recover and learn [10,22]. As suggested by our study participants, we deem the rephrasing suggestions feature helpful in preventing unintentional harm or discouragement of support, even by those experiencing similar oppressions.

Support for encouraging perpetrators or potential allies to rethink their messages can be found in counter speech research [36-38,72]. Unlike content moderation, counter speech does not suppress free expression but aims to reduce hate through persuasion [37]. Counter speech involves 2 main approaches to responding to and deflecting hate speech. The first approach is a nonaggressive response to abusive content, deconstructing stereotypes and misinformation with thoughtful reasoning and fact-bound arguments [38,72]. The second approach, sometimes termed counternarratives, involves creating positive stories to encourage tolerance and disprove the assumptions underlying hate speech [36-38]. Counternarratives focus on triggering positive feelings, such as empathy for targets that could lead to attitude changes [36-38].

Participants were concerned that perpetrators might feel unappreciated and think moderation was unjust or frustrating, potentially leading to retaliation. While participants did not strongly advocate for counter speech explicitly, they thought educational explanations would support users “who have a good heart” [P12] to become allies and adopt rephrasing suggestions. This supports using counternarratives, which aim to foster empathy toward microaggression targets and prompt perpetrators to rethink their actions without feeling wronged.

Furthermore, goals 1 and 5 were influenced by participants’ cultural characteristics, rejecting approaches that would burden their social support network. Participants’ definition of social support includes current allies and microaggression perpetrators who have supportive intentions. Participants noted that the fine line between harmful comments and hard-hearted advice can be hard to identify. Through thoughtful reasoning and positive narratives, counter speech can support unmarried people’s extended support network, such as microaggression perpetrators with supportive intentions. Moreover, counter speech would help perpetrators feel empathy for targets and reconsider their delivery of support, which is currently difficult to achieve for “gray area” moderation cases [30,31].

Goal 4 (ie, having forum-approved experts guide the discussions) offers further insight into who should lead counternarratives. By having forum-approved medical experts and experienced users lead discussions, participants thought counternarratives could help re-evaluate factually wrong, disrespectful, or invalidating content. Leading critical and logical reactions to microaggressions by experienced users is supported by strategies for dealing with racial microaggressions [72]. Eschmann [72] suggested web-based counter spaces—where “activisty” students of color challenge dominant paradigms—highlight and critically react to experiences with microaggressions, providing ample support and counteraction for targets. In these counter spaces, “activisty” students lead critical reactions using counternarratives, similar to the “older sister” [P9] role suggested by our participants.

To nudge authors to revise or delete offensive text, recent work identifies offensive parts of a message [73] and suggests less-offensive alternatives through paraphrasing and style transfer [74,75]. Style transfer aims to translate abusive text into nonabusive while preserving its nonoffensive meaning [33,75,76]. Our study participants also advocated for low-effort approaches. This appreciation of reducing effort for potential allies is supported by other studies on web-based microaggression counteraction strategies [69,73]. Heung et al [69] recommended reactive approaches providing corrective information in response to microaggressions, similar to counternarratives [5,72]. However, participants also expressed concern about making microaggression perpetrators (ie, potential allies) feel unjustly attacked. Thus, public or private rephrasing through style transfer should be further studied to determine whether it could be interpreted as harmful in certain contexts.

Most approaches limit education and explanations to perpetrators, but our participants suggested that all users be educated on microaggressions, how to cope with or address them, and how to find and receive support after experiencing them. They recommended that explanations for flagged comments be made public and include pointers to support groups and resources. They also suggested that educational resources and support groups be added under the microaggression content alert feature for those who do not want to be exposed to microaggressions at all. Thus, social media platforms should extend their educational approaches to benefit *all* users and not just perpetrators.

### Limitations and Future Directions

One potential limitation stems from the interviewer also being an unmarried Korean woman and the study participants being attained using snowball sampling. In investigating the design needs of the population that the interviewer is in and may have weak ties to, the participants may have felt a connection with the interviewer and enabled thoughtful responses. However, they may also have felt pressure to be portrayed as strong, independent women who did not need emotional support, especially when asked how they would alter or not alter their designs if they were the only post author users. Future studies should explore how having a design session facilitator who is not an unmarried Korean woman and does not have weak ties with the participants affects the participants’ designs. In addition, we acknowledge that the findings of our study are based on a small number of participants. Although we reached saturation with 14 participants, the microaggression counteraction designs from this study could still be expanded upon. Thus, we encourage future studies to explore co-designing with a larger number of participants to reach further consensus on the microaggression counteraction designs.

Moreover, we suggest future studies delve into how the proposed microaggression counteraction designs could potentially be weaponized against those who are already susceptible. In this study, the focus was to co-design with unmarried Korean women to counteract the microaggressions suppressing their health care and to explore how the corrosive harm of microaggressions and social and cultural norms were reflected in their needs and preferences. We acknowledge the possibility of weaponization of the microaggression counteraction features. For example, automatically deleting a comment after a set number of negative reactions from the community may easily enable orchestrated actions to take nonharmful comments down. In addition, individualized messages to targets could be used to annoy the target if sent automatically by triggering multiple messages. Furthermore, an expert with a badge who writes microaggressions may encourage higher acceptance of the microaggressions. We suggest subsequent studies investigate how to protect people from the potential weaponization of their designs to ensure their safety.

### Conclusions

In this study, we co-designed with unmarried Korean women participants with the aim to create a resilient and safe space for support on stigmatized SRH by preventing and counteracting web-based microaggressions that suppress women’s health care. During the co-design sessions, we identified 6 goals participants had for counteracting web-based microaggressions that discourage SRH care support seeking. Connecting back to these goals, we also encourage educational, rather than the more common punitive detection and deletion designs that counteract microaggressions. We should help targets and allies cope with microaggressions, and help perpetrators reflect on the consequences of their words. With these design strategies and examples, we hope to encourage new moderation approaches within web-based communities. These approaches should support women in seeking essential care for stigmatized SRH, while reducing the occurrence and detrimental impact of microaggressions on their health and well-being.

## Abbreviations

SRH: sexual and reproductive health
STI: sexually transmitted infection

## Data Availability

The datasets generated and analyzed during this study are not publicly available due to them being transcripts with personally identifiable information and sensitive disclosures about their sexual and reproductive health support–seeking experiences.
